# 

**Published:** 1892-06-04

**Authors:** 


**Notices of Births, Deaths, and Marriages,** are inserted in this column at a charge of 2s. 6d. for an announcement not exceeding four lines.

In Dear Memory of Jacky, aged 10½, who went to Jesus on the 24th May, 1891, and left behind him a loving example of a Christ-like life. Ever remembered by those who knew him. Nurses' Home, Huddersfield. (879)

### NOTICE TO CORRESPONDENTS.

All MS., Letters, Books for Review, and other matters intended for the Editor should be addressed The Editor, The Lodge, Porchester Square, London, W.

The Editor cannot undertake to return rejected M.S., even when accompanied by stamped directed envelope.

**Notice to Advertisers and Subscribers.**

All Advertisements, Orders for Copies of The Hospital, and Business Communications should be addressed to The Publisher, *not to the Editor*, The Hospital, 140, Strand, W.C.

The Cost of Subscription, post free, is as follows:—Per week, 2½d.; per quarter, 2s. 6d.; per half-year, 5s.; per annum, 8s. 6d. Foreign:—Per annum, 10s. 8d.; payable, in all cases, in advance.

**"Burdett's Hospital Annual," 1891—1892.**

Third year of publication. 600 pp. Boards, gilt lettering. A most complete guide to British and Colonial Hospitals, Dispensaries, Nursing and Convalescent Institutions, and Asylums. Price 3s. 6d. Published by The Scientific Press (Limited), 140, Strand, W.C.

**NOTICE.—The Index for Vol. XI. (ending March, 1892) is on sale at the Office of this Journal, price 2½d., post free.**

### SCRAPS AND GLEANINGS.

IT has been proposed to erect an Infectious Diseases Hospital at Windsor.

THE Duke of Rutland has sent a donation of three hundred guineas to the Derby Infirmary.

A FAMILY at Malpas, Cheshire, named Ludlow, have been accidentally poisoned. One person has died.

THE Carlisle Hospital Sunday Fund amounted to £535 0s. 3d., and the Hospital Saturday Fund to £70 9s.

THE Duchess of Portland has issued an appeal on behalf of the East London Hospital for Children at Shadwell.

THE Duchess of Portland will open the new wing of the East London Hospital for Children, at Shadwell, on June 11.

THE bazaar in aid of the building fund of St. Leonards Hospital, Bedford, was opened by the Countess of Limerick.

THE Keighley Town Council now stands virtually pledged to the expenditure of £5,000 for a hospital for infectious diseases.

AT a meeting held recently at Aberdeen it was decided that provision should be made for not less than fifty additional beds to the City Hospital.

THE Children's Country Holidays Fund, of which the subscriptions fell off last year, has received a donation of £250 from Smith's Kensington Charity.

THE Duchess of Connaught last week presented medallions and certificates to 460 members of the St. John's Ambulance Association at Portsmouth.

THE Misses Stokes have given a further donation of £1,500 towards the furnishing ot the Stokes Memorial Convalescent Home at Llanrhos, near Llandudno.

AT the quarterly meeting of the Belfast Royal Hospital it was shown that 200 more intern patients have been treated this year than for the corresponding period of last year.

THE total number of patients treated at the Wigan Infirmary during the past twelve months was 6,137, of this number 1,175 were in-patients and in the children's wards 203 were received.

THE Governors of the District Lunatic Asylum, Kilkenny, have instructed Mr. W. Kaye-Parr, M.A., with a civil engineer of Dublin, to report upon the drainage and water supply of the asylum buildings.

THE Countess of Clanwilliam opened the bazaar in aid of the Convalescent Home for Children, Portsmouth Last year, owing doubtless to some misapprehension the subscriptions to the home amounted to only £60.

THE Victoria Hospital for Children, Chelsea, has received £500 from the London Needlework Guild for the endowment of a Cot presented to Princess May of Teck, and named by her Serene Highness the "Prince Eddy" Cot.

THERE were about 200 guests at the 39th festival dinner of the City of London Hospital for Diseases of the Chest. During the past year the expenditure exceeded the income by £2,848. The Secretary announced subscriptions to the amount of £2,584.

THE Bishop of Carlisle preaching at Workington on Hospital Sunday, said that he had been told that the income of the Workington Infirmary last year had been about £900, and out of that £300 was provided by the pence and half-pence of the working classes.

IT is reported that a proposed gift of £500 from two Birkdale ladies to the New Infirmary, Southport, is likely to be lost because of the refusal of the Committee to insert a clause in the trust deed providing that vivisection will in no case be permitted in the buildings.

THE triennial festival of the supporters of the Royal General Dispensary was held last week. The Lord Mayor presided. According to the report of 1891 there were 13,664 attendances, 4,525 new cases were entered, and 3,578 visits were paid to patients at their own homes.

IT has been decided to hand over the money raised by the Victoria Hall Disaster Fund to the Sunderland Infirmary Committee for the purpose of purchasing, altering, and adapting Heatherstone House, Harrogate, for a convalescent home for children and others belonging to Sunderland.

THE Duchess of Portland opened a bazaar at the Hampstead Conservatoire of Music, Eton Avenue, in aid of the fund for providing a women's ward in the new wing of the Great Northern Central Hospital. This wing is to be erected as a memorial to the late Duke of Clarence and Avondale, who was President of the hospital.

AT a special meeting of the Birkenhead Town Council, it was stated the Health Committee proposed to build immediately four blocks of buildings containing twelve beds each and one block containing eight beds for isolation purposes or for paying patients, together with the laundries, mortuary, and other necessary rooms.

### BOOKS RECEIVED.

Batsford and Co.

"House Drainage." C. Middleton.

George Bell,

"Camping Out." By A. Macdonell, M.A.

Longmans and Son.

"Dissection Guides." By Thomas Cooke, F.R.C.S.

Bailliere and Co.

"How to Feel the Pulse." W. Ewart, M.D.

"The Pocket Case Book." By A. T. Brand, M.D.

T. and A. Constable.

"Atlas of Clinical Medicine." Vol. 1, Part IV. By Byrom Bramwell, M.D., &c.

Methuen and Co.

"Air and Water." Vivian B. Lewes.

### THE STUDENT OF MEDICINE.

[All communications for this Supplement should be addressed to THE EDITOR of THE HOSPITAL, at 140, Strand, with the words, "Students' Column," written in the left hand top corner of the envelope.]

### APPOINTMENTS.

Adams, E. W., M.R.C.S., L.R.C.P., appointed Assistant Accoucheur to King's College Hospital, Lincoln's Inn Fields. Andrew, J. Grant, M.B., C.M.Glasg., appointed Dispensary Surgeon to the Glasgow Victoria Infirmary. Barnett, Lawrence, M.D.Lond., M.R.C.S., appointed Honorary Physician to the Kilburn, Maida Vale, and St. John's Wood General Dispensary. Berthon, Ellen, M.B.Lond., appointed Physician to the Out-Patient Department (Wandsworth Road) of Clapham Maternity Hospital, London. Blake, S. R., M.R.C.S., L.R.C.P.Lond., appointed Senior House-Surgeon to the West Ham Hospital. Dempster, J. H., M.R.C.S., L.R.C.P., appointed Accoucheur to King's College Hospital, Lincoln's Inn Fields. Ferguson, George G., M.B., C.M.Glasg., appointed Assistant Medical Officer to the Convalescent Branch of St. John's Hospital for Diseases of the Skin. Fisher, T. E., L.R.C.P.Lond., M.R.C.S., appointed House Surgeon to the West Norfolk and Lynn Hospital, Lynn. Godson, J. E., L.R.C.P.Lond., M.R.C.S., appointed Assistant Medical Officer to the Birmingham Workhouse Infirmary. Hamper, Chas. N., M.B. and C.M.Edin., appointed Assistant Resident Medical Officer to the North-West London Hospital. Hillier, R. J., M.R.C.S., L.R.C.P.Lond., appointed Junior House Surgeon to the West Ham Hospital. Hughes, E. Lucas, M.R.C.S.Eng., L.R.C.P.Lond., appointed Junior Obstetric Assistant at University College Hospital. Jäger, H. J., M.R.C.S., L.R.C.P., appointed House Surgeon to King's College Hospital, Lincoln's Inn Fields. Lister, Sir Joseph, Bart., M.B. Lond., F.R.C.S.Eng., F.R.S., appointed Honorary Consulting Surgeon to the Royal Ear Hospital, Soho. Marnoch, John, M.B., C.M.Aberd., appointed House Physician and Surgeon to the Sick Children's Hospital, Aberdeen. Pritchard, Erich, M.B. M.R.C.S., L.R.C.P., appointed House Physician to King's College Hospital, Lincoln's Inn Fields. Ramsay, George, M.R.C.S., L.R.C.P., appointed House Surgeon to King's College Hospital, Lincoln's Inn Fields. Smith, J. Waddelow, L.S.A., appointed Clinical Ophthalmic Assistant to King's College Hospital, Lincoln's Inn Fields. Smith, W. R., L.S.A., appointed Assistant House Physician to King's College Hospital, Lincoln's Inn Fields. Sutherland, Chas. D., M.B. and C.M.Edin., appointed Resident Medical Officer to the North-West London Hospital, *vice* C. H. Fowler, M.R.C.S., L.R.C.P., resigned. Waddelow, John J., M.R.C.S., L.R.C.P., L.S.A., appointed House Surgeon to King's College Hospital, Lincoln's Inn Fields. West, John G. U., L.R.C.P.Lond., M.R.C.S.Eng., appointed Ophthalmic Surgeon to the North Staffordshire Infirmary, Stoke-on-Trent, *vice* W. H. Folker, F.R.C.S.Eng., resigned. Wilson, Ann A., M.D.Brux., L.R.C.P. and S.Edin., appointed Resident Medical Officer to the Clapham Maternity Hospital, London.

### VACANCIES.

### Resident Medical Officers.

The following vacancies are announced this week, the amount of salary in each case being given after the name of the institution:

Great Yarmouth Hospital, House Surgeon, doubly qualified—Salary £90 per annum, with board, &c.

Royal Free Hospital, Gray's Inn Road, Junior Resident Medical Officer, doubly qualified.—Board, &c.

Salop Infirmary, Shrewsbury, House Surgeon, doubly qualified—Salary £100 per annum, with board, &c.

Taunton and Somerset Hospital, Taunton, Assistant House Surgeon; appointment for six months—Board, &c.

Wilts County Asylum, Devizes, Assistant Medical Officer, unmarried—Salary £120 per annum, with an annual increase to £150, with board, &c.

Worcester General Infirmary, Assistant to House Surgeon; qualified, unmarried; to act also as Dispenser—Salary £70 per annum, with board, &c.

### Non-Resident Medical Officers.

Chelsea Hospital for Women, Fulham Road, S.W., Clinical Assistants.
Dental Hospital of London, Leicester Square, W.C., Dental Surgeon.
Dental Hospital of London and London School of Dental Surgery, Leicester Square, Demonstrator—Honorarium £50 per annum.
Liverpool Royal Infirmary, Assistant Physician.
Liverpool Royal Infirmary, Two Assistant Honorary Surgeons.
North Staffordshire Infirmary and Eye Hospital, Hartshill, Stoke-on-Trent, Assistant Ophthalmic Surgeon.
Wolverhampton and District Hospital for Women, two Honorary Acting Gynæcological Surgeons.

### PASS LISTS.

### University of London.

M.B. PASS EXAMINATION.—MAY, 1892.

FIRST DIVISION.—A. N. Boycott, St. Thomas's Hospital; H. Caiger, University College; H. Finley, University College; M. L. Hepburn, St. Bartholomew's Hospital; T. Holmes, Guy's Hospital; W. K. Hughes, St. Bartholomew's Hospital; H. L. Lack, King's College; P. Lord, Guy's Hospital; J. H. Parsons, B. Sc., Medical School, Bristol, and University College; A. W. Sheen, Guy's Hospital; Caroline Sturge, London School of Medicine for Women; W. A. Sturge, London Hospital; A. L. Whitehead, Yorkshire College.

SECOND DIVISION.—A. J. Adkins, St. Thomas's Hospital; Annie M. S. Anderson, London School of Medicine, and Royal Free Hospital; W. J. Cameron, Queen's College, Belfast; C. J. Girling, Guy's Hospital; J. W. F. Jewell, Guy's Hospital; S. B. Mitra, B. Sc., University College; W. B. Morton, University College; Amy Sheppard, London School of Medicine, and Royal Free Hospital; W. R. Smith, King's College; W. K. Walls, Owens College, and Manchester Royal Infirmary.

### Royal College of Surgeons of England,

The following gentlemen having passed the necessary examinations and having conformed to the By-laws and Regulations, were at the ordinary meeting of the Council admitted Members of the College: M. F. Agar, L.R.C.P., J. B. Anderson, L.R.C.P., W. J. Ansorge, L.R.C.P., H. C. Arathoon, L.R.C.P., S. S. Argyle, L.R.C.P., J. A. Atkinson, L.R.C.P., R. T. Bakewell, L.R.C.P., J. I. Barberi, L.R.C.P., *W. R. M. Berridge, L.R.C.P., W. E. F. Bird, L.R.C.P., G. T. Birdwood, L.R.C.P., W. Bligh, L.R.C.P., *A. O. Bobardt, M.B.Melb., W. A. Bowring, L.R.C.P., A. B. Boyd, L.R.C.P., *W. H. Bracewell, M.B.Melb., E. Bromhall, L.R.C.P., S. M. Brown, L.R.C.P., G. D. Browning. L.R.C.P., W. Bubb, L.R.C.P., *H. L. Cade, L.R.C.P., A. M. Campbell, L.R.C.P., H. L. Carre-Smith, L.R.C.P., W. F. Colclough, L.R.C.P., J. T. H. Croft, L.R.C.P., E. L. Cropp, L.R.C.P., A. V. Crossing, L.R.C.P., H. J. Cummings, L.R.C.P., R. N. Daniel, L.R.C.P., G. C. Davies, L.R.C.P., W. A. Dow, L.R.C.P., F. E. Easton, L.R.C.P., C. C. Elliott, L.R.C.P., A. Emery, L.R.C.P., J. R. Evans, L.R.C.P., *W. M. Fisher, L.S.A., M. B. Foster, L.R.C.P., E. E. Frazer, L.R.C.P., C. A. Fuller, L.R.C.P., A. Y. Fullerton, L.R.C.P., J. B. Garman, L.R.C.P., A. W. German, L.R.C.P., G. V. M. Gideon, L.R.C.P., B. B. Gilpin, L.R.C.P., B. W. N. Gowring, L.R.C.P., *S. D. Graham, L.R.C.P., C. W. Grant, L.R.C.P., E. H. Greaves, L.R.C.P., P. Gross, L.R.C.P., J. Hake, L.R.C.P., L. L. Hanham, L.R.C.P., *A. B. Harman, L.S.A., C. H. Harcourt, L.R.C.P., W. J. Harper, L.R.C.P., J. E. Harris, L.R.C.P., A. G. Harvey, L.R.C.P., J. Harvey, L.R.C.P., J. O. Harvey, L.R.C.P., H. L. Hatch, L.R.C.P., A. F. Heaton, L.R.C.P., H. D. Hey, L.R.C.P., E. Hill, L.R.C.P., J. P. Hocken, L.R.C.P., E. L. Hughes, L.R.C.P., E. P. Isaacs, L.R.C.P., R. H. Jackson, L.R.C.P., H. J. Jäger, L.R.C.P., E. W. James, L.R.C.P., *H. H. A. Jones, L.R.C.P., H. J. R. Jones, L.R.C.P., B. C. Kendall, L.R.C.P., G. Kendrick, L.R.C.P., J. C. King, L.R.C.P., E. E. B. Landon, L.R.C.P., A. J. Lattey, L.R.C.P., *D. C. M. Lunt, L.K.Q.C.P.I., W. D. Loveday, L.R.C.P., E. J. MacGrath, L.R.C.P., K. M. Mackenzie, L.R.C.P., M. M. Makalua, L.R.C.P., E. G. March, L.R.C.P., G. Master, L.R.C.P., P. M. May, L.R.C.P., G. M. Mellor, L.R.C.P., W. B. Mercer, L.R.C.P., W. H. Morgan, L.R.C.P., C. G. F. Morrice, L.R.C.P., F. T. Morris, L.R.C.P., C. K. Moseley, L.R.C.P., H. P. Motteram, L.R.C.P., F. S. Noble, L.R.C.P., H. FitzS. Nunes, L.R.C.P., T. Olver, L.R.C.P., W. H. Orr, L.R.C.P., A. Paling, L.R.C.P., W. M. Palmer, L.R.C.P., R. McI. Paton, L.R.C.P., G. A. Peake, L.R.C.P., E. B. Pickthorn, L.R.C.P., J. R. R. Pollock, L.R.C.P., F. A. Pond, L.R.C.P., *W. Porritt, L.K.Q.C.P.I., E. Prada, L.R.C.P., D. C. Rayner, L.R.C.P., H. K. Rayson, L.R.C.P., A. W. Read, L.R.C.P., J. H. Rivers, L.R.C.P., C. A. Ryde, L.R.C.P., F. G. Saffery, L.R.C.P., A. S. St. John, L.R.C.P., *T. G. Scott, L.R.C.P., E. W. Senior, L.R.C.P., D. F. Shearer, L.R.C.P., A. W. Sheen, L.R.C.P., G. T. S. Sichel, L.R.C.P., D. Sims, L.R.C.P., A. A. Smith, L.R.C.P., W. W. Smith, L.R.C.P., D. Stead, L.R.C.P., F. C. Stearn, L.R.C.P., E. A. Sworn, L.R.C.P., P. O. Thompson, L.R.C.P., J. A. Thyne, L.R.C.P., T. E. Tibbets, L.R.C.P., J. P. Tildesley, L.R.C.P., H. E. Tracey, L.R.C.P., A. E. E. Twynam, L.R.C.P., R. R. M. Walthard, L.R.C.P., S. W. C. Warneford, L.R.C.P., R. L. Wason, L.R.C.P., G. W. B. Waters, L.R.C.P., J. A. W. Watts, L.R.C.P., A. N. Weir, L.R.C.P.; H. W. West, L.R.C.P., *P. S. B. Wetherall, L.R.C.P., C. E. Wheeler, L.R.C.P., S. L. B. Wilkes, L.R.C.P., J. Williamson, L.R.C.P., A. M. Wilson, L.R.C.P., W. C. Wright, L.R.C.P.

* Candidates who have not presented themselves under the regulations of the Examining Board in England.

### Royal College of Surgeons in Ireland.

FELLOWSHIP EXAMINATIONS. — The following gentlemen, having passed the necessary examinations, have been admitted Fellows of the College: Mr. Edward Churchill Stack, Mr. Crawford Warren, Mr. William Henry Edward Woodwright, and Mr. Henry Benson Goulding.

